# Haplotyping-based preimplantation genetic testing reveals parent-of-origin specific mechanisms of aneuploidy formation

**DOI:** 10.1038/s41525-021-00246-0

**Published:** 2021-10-07

**Authors:** Olga Tšuiko, Michiel Vanneste, Cindy Melotte, Jia Ding, Sophie Debrock, Heleen Masset, Maire Peters, Andres Salumets, Anne De Leener, Céline Pirard, Candice Kluyskens, Katleen Hostens, Arne van de Vijver, Karen Peeraer, Ellen Denayer, Joris Robert Vermeesch, Eftychia Dimitriadou

**Affiliations:** 1 Department of Human Genetics, Centre for Human Genetics, University Hospitals Leuven, Leuven, 3000 Belgium; 2 Laboratory of Cytogenetics and Genome Research, Centre for Human Genetics, KU Leuven, Leuven, 3000 Belgium; 3 Leuven University Fertility Center, University Hospitals Leuven, Leuven, 3000 Belgium; 4 Department of Obstetrics and Gynaecology, Institute of Clinical Medicine, University of Tartu, Tartu, 50406 Estonia; 5 Centre for Human Genetics, Cliniques Universitaires Saint Luc, UCLouvain, Brussels, 1200 Belgium; 6 Department of Gynaecology, Cliniques Universitaires Saint Luc, UCLouvain, Brussels, 1200 Belgium; 7 Centre for Reproductive Medicine (CRG)-Brugge-Kortrijk, AZ Sint-Jan Brugge-Oostende AV, Brugge, 8000 Belgium

**Keywords:** Genomic instability, Development

## Abstract

Chromosome instability is inherent to human IVF embryos, but the full spectrum and developmental fate of chromosome anomalies remain uncharacterized. Using haplotyping-based preimplantation genetic testing for monogenic diseases (PGT-M), we mapped the parental and mechanistic origin of common and rare genomic abnormalities in 2300 cleavage stage and 361 trophectoderm biopsies. We show that while single whole chromosome aneuploidy arises due to chromosome-specific meiotic errors in the oocyte, segmental imbalances predominantly affect paternal chromosomes, implicating sperm DNA damage in segmental aneuploidy formation. We also show that postzygotic aneuploidy affects multiple chromosomes across the genome and does not discriminate between parental homologs. In addition, 6% of cleavage stage embryos demonstrated signatures of tripolar cell division with excessive chromosome loss, however hypodiploid blastomeres can be excluded from further embryo development. This observation supports the selective-pressure hypothesis in embryos. Finally, considering that ploidy violations may constitute a significant proportion of non-viable embryos, using haplotyping-based approach to map these events might further improve IVF success rate.

## Introduction

Chromosomal anomalies are common in human natural conception, resulting in early pregnancy loss or congenital disorders in newborns. Chromosome missegregations during gametogenesis result in zygotic inheritance of meiotic aneuploidy. Postzygotic mitotic aneuploidy can affect all cells when it occurs during the first zygotic division or a subset of cells at later cellular divisions. Consequently, postzygotic mitotic errors lead to the formation of blastomeres with different genomic constitution^1–5^. In the last decade, many studies on human preimplantation embryos have been carried out using a multitude of techniques to examine the frequency of chromosomal aneuploidy and its impact on embryo development^6–9^. Most of these studies were performed under the framework of preimplantation genetic testing for aneuploidy (PGT-A). However, technologies such as array comparative genomic hybridization (aCGH) or low-pass next generation sequencing (NGS), which are routinely used for PGT-A, do not provide information on the mechanistic or parental origin of aneuploidy nor allow to map uniparental disomy (UPD) or genome-wide ploidy anomalies. In addition, PGT-A is mainly offered to IVF patients with fertility issues, and some of the IVF indications can influence embryo aneuploidy rate^10–12^. Therefore, the true genomic landscape of human embryos in the general population remains elusive.

The development of genome-wide haplotyping methods for preimplantation genetic testing of monogenic disorders (PGT-M), such as karyomapping^13^, siCHILD/haplarithmisis^14^ or OnePGT^15^, allowed to gain deeper understanding of genetic abnormalities in human embryos^16–18^. In parallel to haplotyping-based mutation analysis, these assays allow to characterize a wide range of chromosome aberrations across the genome to which conventional PGT-A methods are blind. One of the most comprehensive studies so far investigated the genomic profiles of approximately 1000 IVF embryos, derived from PGT-M couples, by applying karyomapping on trophectoderm (TE) biopsies^19^. The study characterized the parental origin and frequency of various genomic abnormalities in blastocysts, including whole and segmental meiotic aneuploidy, UPD, triploidy and haploidy.

Here, we complement the existing knowledge by performing a comprehensive genomic assessment of cleavage-stage embryos and blastocysts from a large haplotyping-based PGT cohort. In contrast to PGT-A, our cohort mainly consists of PGT-M patients, the majority of which are relatively young and presumably fertile, and they undergo assisted reproduction to avoid the transmission of a hereditary genetic disorder to their offspring. We mapped the incidence and nature of common and rare abnormalities, spanning early human development. By using siCHILD/haplarithmisis, we inferred both parent-of-origin and meiotic or mitotic signatures of chromosome missegregations to understand the underlying mechanisms of aneuploidy formation. Together, these data provide a comprehensive view of genomic anomalies found in human embryos at different stages of preimplantation development, which can further provide insight for guiding embryo selection strategies.

## Results

### Distribution of distinct types of genomic aberrations in cleavage-stage embryos and blastocysts

**Fig. 1:**
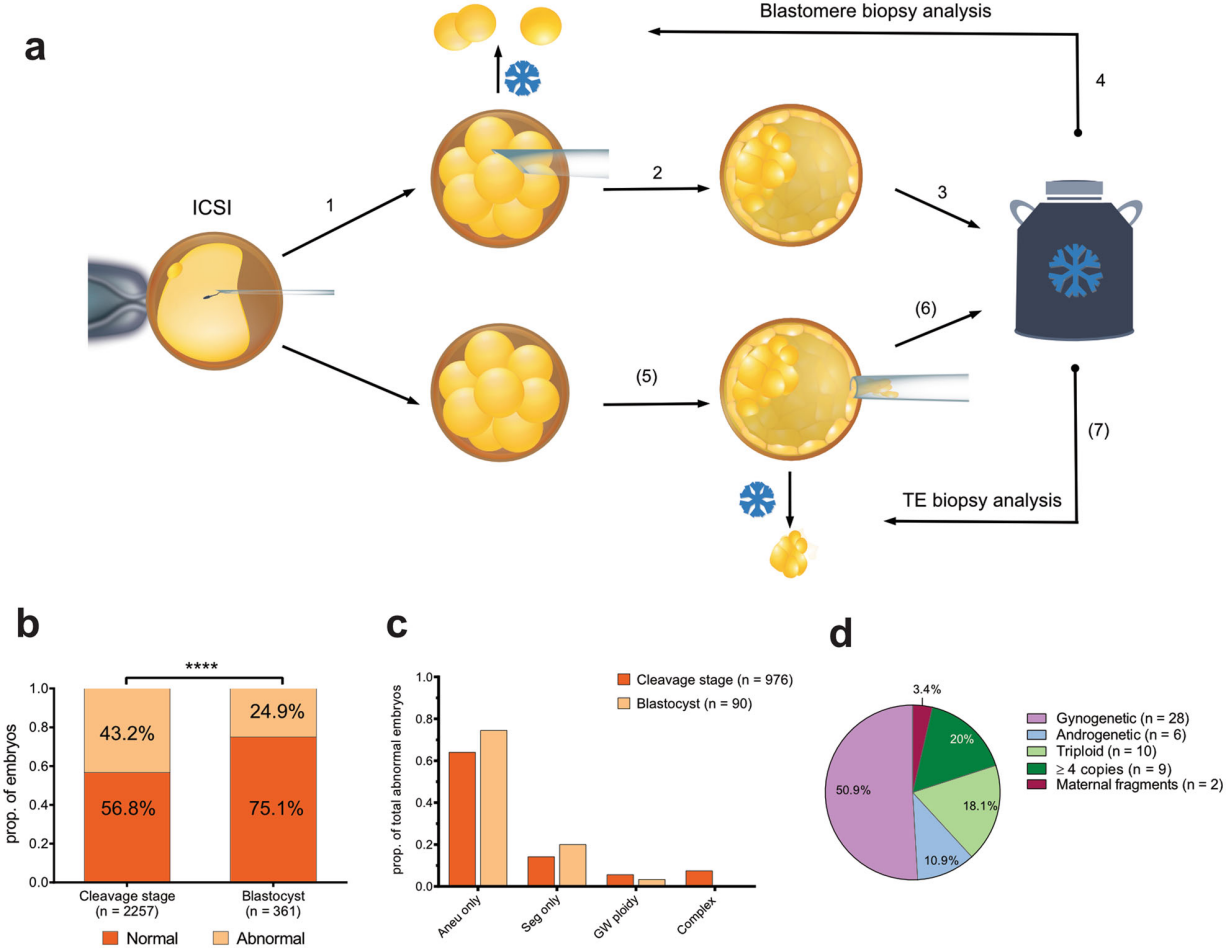
Genomic landscape of cleavage stage embryos and blastocysts derived from PGT-M patients. **a** Following intracytoplasmic sperm injection (ICSI), one or two blastomeres are biopsied on day-3 (D3) (1). Following D3 biopsy, embryos are cultured further until blastocyst (2) and are cryopreserved (3). Biopsied blastomeres were analysed only following successful embryo cryopreservation (4). If cleavage stage embryo fails to develop further after the biopsy, the biopsied material was not analysed (except for a subset of D3 embryos that were processed immediately). Alternatively, trophectoderm (TE) biopsy was performed, followed by blastocyst cryopreservation and TE biopsy analysis (5–7). **b** Frequency of abnormal cleavage embryos and blastocysts (two-tailed Fisher exact test, ****P < 0.0001). **c** Distribution of various genomic aberrations in abnormal embryos (aneu aneuploidy, seg segmental imbalances, GW genome-wide). **d** Genome-wide ploidy aberrations in cleavage embryo biopsies.

To characterize the genome of preimplantation embryos, we retrieved 2778 biopsies, derived from 2706 embryos, and analysed them retrospectively. The maternal age at the start of first PGT cycle ranged from 22 to 42, with the mean of 30.11 (±3.95). According to the currently existing haplotyping-based PGT workflow (Fig. 1a), when a day-3 (D3) biopsy is performed, it will be analysed only if the embryo develops further into a blastocyst and is cryopreserved (except for 180 D3 biopsies from 24 families that were analysed immediately). Alternatively, TE biopsy was performed on day 5/6. In total, 95.8% of biopsies were informative (*n* = 2661/2778), originating from 2618 embryos: 2300 biopsies were derived from 2257 cleavage-stage embryos (n = 2257/2618, 86.2%) and 361 were TE biopsies performed at blastocyst stage (*n* = 361/2618, 13.8%). The incidence of genomic aberrations was significantly higher in cleavage embryos (*n* = 976/2257, 43.2%) compared with blastocysts (*n* = 90/361, 24.9%; *P* < 0.0001, two-tailed Fisher’s exact test) (Fig. 1b). For blastocysts, the mosaicism rate based on the analysis of a single TE biopsy was 9.4%, which falls in the range of previously reported rates, varying from 2 to 13%^20–22^. Whole chromosome aneuploidy was the most frequently observed event, and in most cases, aneuploidy was the sole abnormality within an embryo, affecting 64 % and 74.4% of abnormal D3 and D5/6 embryos, respectively (Fig. 1c). Segmental imbalances mostly occurred as an isolated event in blastocysts, but in cleavage embryos they were often accompanied by whole chromosome aneuploidy. In 15 embryos, derived from two families, segmental imbalances were a result of a balanced translocation in one or both parents. This accounted for approximately 10% of all embryos with isolated segmental aberrations. In addition, we detected 73 D3 embryos (7.5%, *n* = 976) with complex abnormal biopsy profiles that had full or segmental chromosome losses and gains, UPDs and/or nullisomies across the genome. The presence of various distinct chromosome abnormalities across the whole genome did not allow to determine the precise copy number in complex abnormal embryos due to logR normalization issues. Hence, these embryos were excluded from further analysis.

### Uniform genome-wide ploidy aberrations in early embryo development

Genome-wide ploidy violations, such as haploidy/genome-wide UPD and triploidy were detected in 2.4% and 0.8% of all D3 and TE biopsies, respectively. We classified embryos as gynogenetic (carrying only maternal DNA) or androgenetic (carrying only paternal DNA), and triploid embryos as digynic or diandric, respectively. By using haplotype information, we also discriminated meiosis I (MI) and meiosis II (MII) errors from mitotic events that occur during zygotic/postzygotic division (Supplementary Fig. S1). From D3 embryos with genome-wide anomalies, half carried gynogenetic blastomeres (*n* = 28, Fig. 1d). Two blastomeres also had only maternal chromosome fragments. In contrast, androgenetic cells with solely paternal chromosomes were detected in six embryos. The remaining D3 biopsies had a gain of extra set(s) of chromosomes, resulting in triploidy (*n* = 10), tetraploidy (*n* = 8), or polyploidy (*n* = 1). All triploid blastomeres were digynic in origin: six triploidies arose due MI or MII errors in the oocyte, whereas the observed haplotypes of other four embryos indicated a potential mitotic error. From tetraploid cells four were trigynic, arising due to MI (*n* = 3) or mitotic error (*n* = 1) and four were balanced tetraploid (2pat:2mat alleles) with additional chromosome missegregations. Generation of balanced tetraploid blastomeres is suggestive of cytokinetic failure or blastomere fusion during first postzygotic divisions^23^. In blastocysts, uniform genome-wide anomalies were present only in three of all TE biopsies: one digynic triploid of meiotic origin (MI error), a digynic triploid of mitotic origin and likely a parthenogenetic embryo with retained 2nd polar body (Supplementary Fig. S2).

### Parental and mechanistic origin of aneuploidy in human embryos

**Fig. 2:**
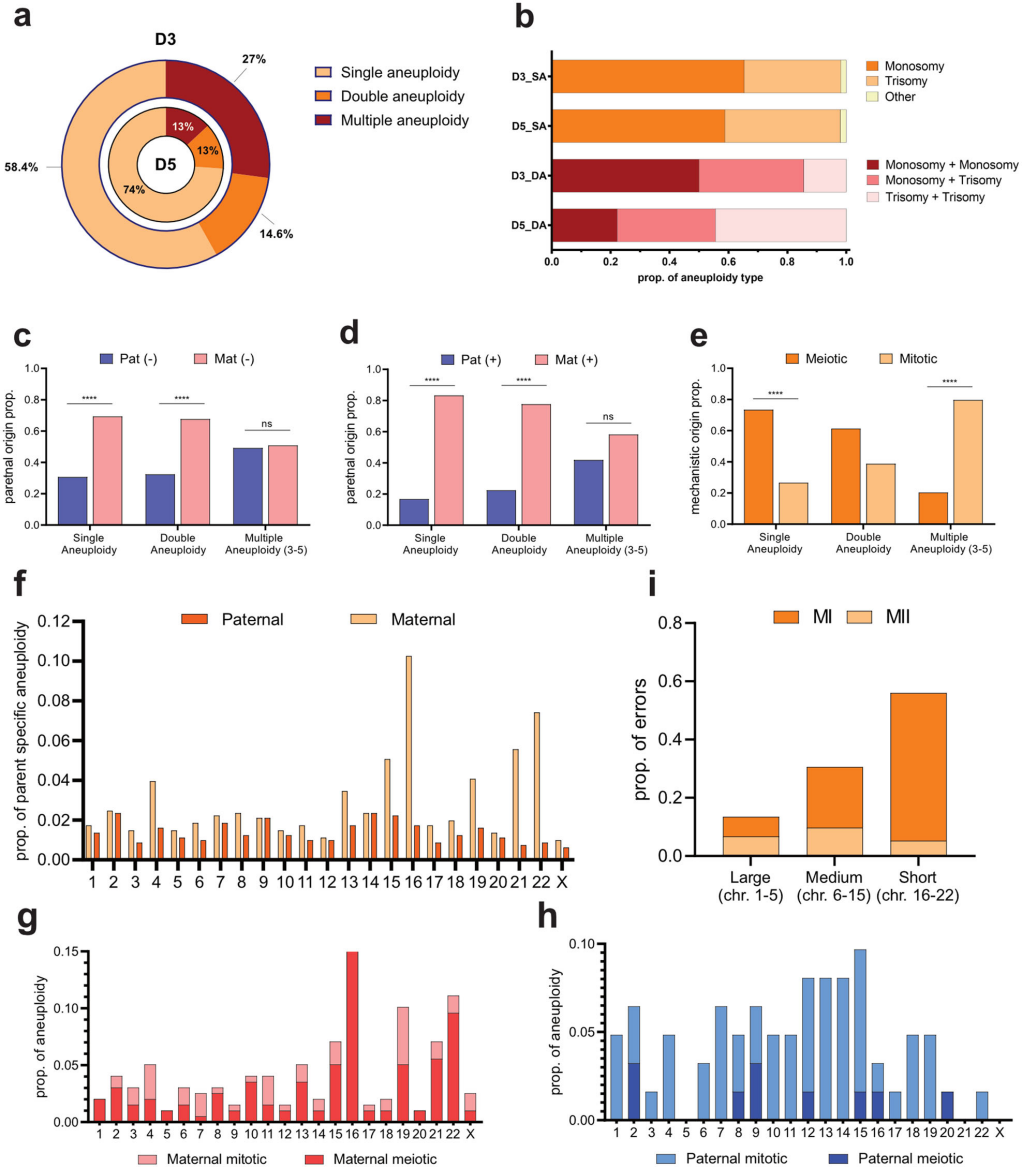
Origin and chromosome-specific rate of aneuploidy in blastomeres of human IVF embryos. **a** Frequency of single, double and multiple (3–23 chromosomes affected) aneuploidy in cleavage embryos (D3) and blastocysts (D5) (*n* = 712 and *n* = 69, respectively). **b** Proportion of aneuploidy types in single (SA) and double (DA) aneuploidy in cleavage embryos (D3, *n* = 416 and 104, respectively) and blastocysts (D5, *n* = 53 and 9, respectively). **c** Parental origin of monosomy in cleavage embryos; *n* = 274, 140, and 122 for single, double and multiple (3–5 chromosomes affected) aneuploidy, respectively (two-tailed binomial test, ****P < 0.0001). Parental (**d**) and mechanistic (**e**) origin of trisomy in cleavage embryos; *n* = 134, 68, and 67 for single, double and multiple (3–5 chromosomes affected) aneuploidy, respectively (two-tailed binomial test, ^****^P < 0.0001). **f** Rate and distribution of paternal and maternal aneuploidy (*n* = 805) across the genome in cleavage embryos. Rate and mechanistic origin of maternal (*n* = 199) (**g**) and paternal (*n* = 62) (**h**) trisomy across the genome in cleavage embryos. **i** Association between maternal meiotic error type and chromosome classification by length (*n* = 139, *P* < 0.0001, *χ*² for trend).

To unravel the mechanisms of aneuploidy formation, we classified different aneuploidy patterns in both blastomeres and TE biopsies. Single aneuploidy was the most common type in both D3 and D5/6 embryos (Fig. 2a). We observed a prevalence of single monosomy over single trisomy (*n* = 408, *P* < 0.0001, binomial test) in cleavage embryos (Fig. 2b), but no such difference was evident in blastocysts (*n* = 53, *P* = ns). Although our TE data was scarce, this result is in line with previous large-scale analysis of TE embryo biopsies, showing similar rate of chromosome losses and gains at blastocyst stage^11,24^. Rare chromosomal abnormalities, such as UPD (*n* = 2), >4 copies (*n* = 5) and isolated nullisomy (*n* = 1), were observed in 2% of all single aneuploidies in D3 embryos; and one potential mosaic UPD was also detected in TE biopsy. When looking at double aneuploidy, chromosome losses also seemed to be more prevalent than gains in D3 embryos via monosomy:monosomy or monosomy:trisomy combinations (Fig. 2b). Various aneuploidy patterns were equally distributed in TE biopsies, however blastocysts with double aneuploidy were rare in our dataset (*n* = 9).

As genome-wide haplotyping allows to determine the parental and mechanistic origin of different aneuploidy types, we characterized the nature of aneuploidies in our cohort. When only one or two chromosomes were involved, the parental origin of aneuploidy was mainly maternal, demonstrating at least a 2-fold increase in the number of affected maternal chromosomes in both monosomies and trisomies (*P* < 0.0001, binomial test; Fig. 2c, d). Given that single chromosome missegregations are associated with meiotic errors in the oocyte^11^, the bias towards maternal chromosomes coincides with the observed mechanistic origin of trisomies, as more than half had signatures of maternal MI or MII gain in D3 embryo (*n* = 134, *P* < 0.0001, binomial test; Fig. 2e). Considering that chromosome-specific monosomy and trisomy rates were similar, most maternal chromosome losses can likely be attributed to meiotic chromosome missegregation in the oocyte. In contrast, when more chromosomes are affected by aneuploidy, postzygotic mitotic chromosome missegregations become the dominant form of aneuploidy formation that does not discriminate between maternal and paternal homologs (Fig. 2c–e). For blastocysts, the mitotic origin of some of the monosomies can be distinguished from meiotic events by the presence of chromosomal mosaicism. However, the correlation between the parental and mechanistic origin, which was observed in D3 biopsies, is not evident in TE biopsies due to the limited number of aneuploid blastocysts (Supplementary Fig. S3).

We next looked whether the distinct aneuploidy rate could vary amongst chromosomes. To map the distribution of nondisjunction-based aneuploidy across the genome, we only included embryos with ≤5 affected chromosomes. We observed a large variation in aneuploidies amongst the different chromosomes, however most affected chromosomes were 15, 16, 19, 21, and 22 (Fig. 2f). Paternal aneuploidy was equally distributed across different chromosomes. In contrast, maternal aneuploidy showed chromosome-specific susceptibility to aneuploidy. This difference in the parent-of-origin also mirrors the mechanism of aneuploidy formation: paternal trisomies arise during postzygotic divisions, while maternal trisomies are mainly meiotic in origin with a strong bias towards certain chromosomes (Fig. 2g, h). By looking at haplotypes, we determined that chromosome non-disjunction in MI accounted for 80% of all maternal meiotic errors (*n* = 142). The rate and type of meiotic errors in the oocyte seems to depend on chromosome length and classification, as shorter and/or acrocentric chromosomes had a higher rate of MI errors (*n* = 139, *P* < 0.0001, *χ*² for trend; Fig. 2I). This effect was also present, when trisomy 16 was excluded, as it accounted for almost 1/4 of all maternal meiotic aneuploidies (*n* = 108, *P* = 0.005, *χ*² for trend). When we analysed TE biopsies, chromosomes 15, 16, 21, and 22 were also commonly observed; however, there was no specific trend in aneuploidy type and distribution due to limited amount of data (Supplementary Fig. S4). Therefore, more studies on blastocysts are warranted to characterize the chromosome-specific distribution of parental and mechanistic origins of aneuploidy at later stages of development.

### Hypodiploid blastomeres arrest in development following tripolar division

**Fig. 3:**
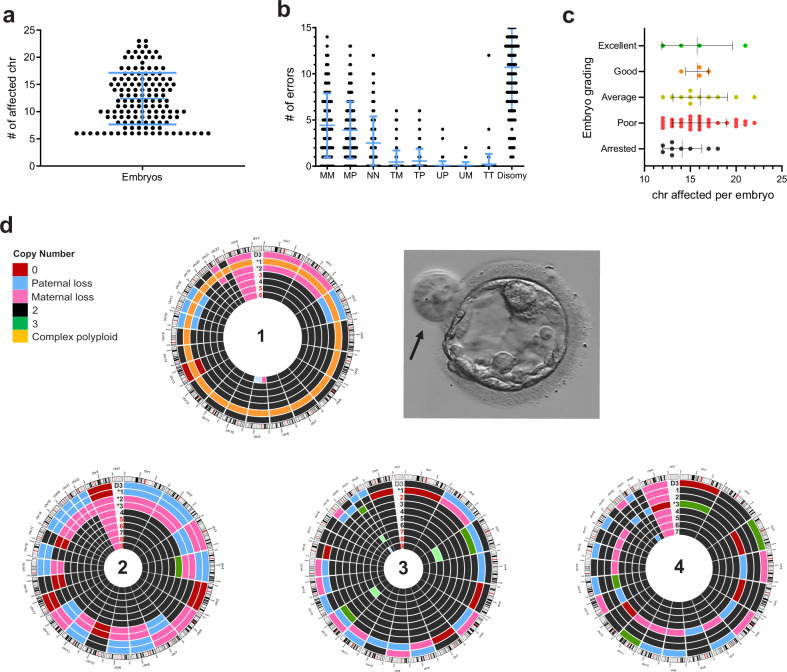
Hallmarks of putative tripolar division in cleavage embryos and blastocysts. **a** Each dot represents an embryo (*n* = 138) with corresponding number of affected chromosomes in the genome, with a mean of 12.41 ± 4.74 (±SD) affected chromosomes per embryo (chromosomes = 23). **b** Rate of different chromosome missegregations in embryos with multiple aneuploidy (MM monosomy maternal, MP monosomy paternal, NN nullisomy, TM trisomy maternal, TP trisomy paternal, UM maternal UPD, UP paternal UPD, TT tetraploidy). **c** Developmental fate of embryos with putative tripolar division (Arr arrested, EB early blastocysts, Good good-quality blastocyst, Poor poor-quality blastocyst). **d** Circus plots representing genomic constitution of four blastocysts, derived from cleavage embryos with putative tripolar division. Results of day-3 biopsy are indicated as D3. Asterisk indicates large potentially excluded cells (as those indicated by arrow in Embryo 1), compared to other cells in the blastocyst. Numbers in red in Embryos 1, 2, and 3 indicate that a clump of cells (on average 2–4 cells) was tubed together and analysed. Light green (in Embryo 3) and light blue (in Embryo 1) indicate mosaic gain and loss, respectively.

In contrast to blastocysts, we observed that cleavage embryos also contained cells with multiple aneuploidies, affecting more than five chromosomes, with a mean of 12.41 ± 4.74 (±SD) (Fig. 3a). This result is suggestive of putative tripolar cell division, which causes chromosomes to segregate across three daughter blastomeres, each inheriting ~31 chromosomes per cell^25^. The observed ratio of disomies, maternal monosomies, paternal monosomies and nullisomies in these embryos (4.97:2.05:1.82:1.16, respectively) was similar to the predicted 4:2:2:1 ratio of tripolar division, which generates hypodiploid complements with excess chromosome loss (Fig. 3b). Tripolar mitosis often leads to embryonic arrest prior to morula formation^26,27^, possibly due to the disruption of embryonic genome activation (EGA) that drives preimplantation development beyond cleavage-stage^28,29^. In our cohort most cleavage embryos with hypodiploid blastomeres developed into blastocysts of variable quality, although majority were of poor morphological grading (Fig. 3c and Supplementary Table S1). Thus, we hypothesized that putative tripolar division might have occurred at the 2-cell or 4-cell stage rather than during the first zygotic division, creating a mixture of normal and aberrant cells.

To test this hypothesis, we analyzed individual blastomeres of four blastocysts derived from cleavage embryos with signatures of tripolar division. Indeed, this analysis identified both normal and aberrant cells in all analyzed blastocysts (Fig. 3d). The identical, reciprocal, or highly similar profile of hypodiploid cells to the day-3 biopsy also proves their clonal origin. Furthermore, based on the morphology of blastocysts, hypodiploid or complex abnormal blastomeres were evidently larger compared to other cells, and in one embryo they were excluded from blastocyst formation (Fig. 3d). The presence of such cells was seen in approximately 30% of blastocyst that had hypodiploid blastomeres at day-3 (Supplementary Table S1). These results suggest that following tripolar mitosis, further proliferation of abnormal cells is blocked at day-3 of development, while normal blastomeres continue their division, ensuring embryo growth, and survival.

### Characterization of segmental aneuploidy in D3 and D5 embryos

**Fig. 4:**
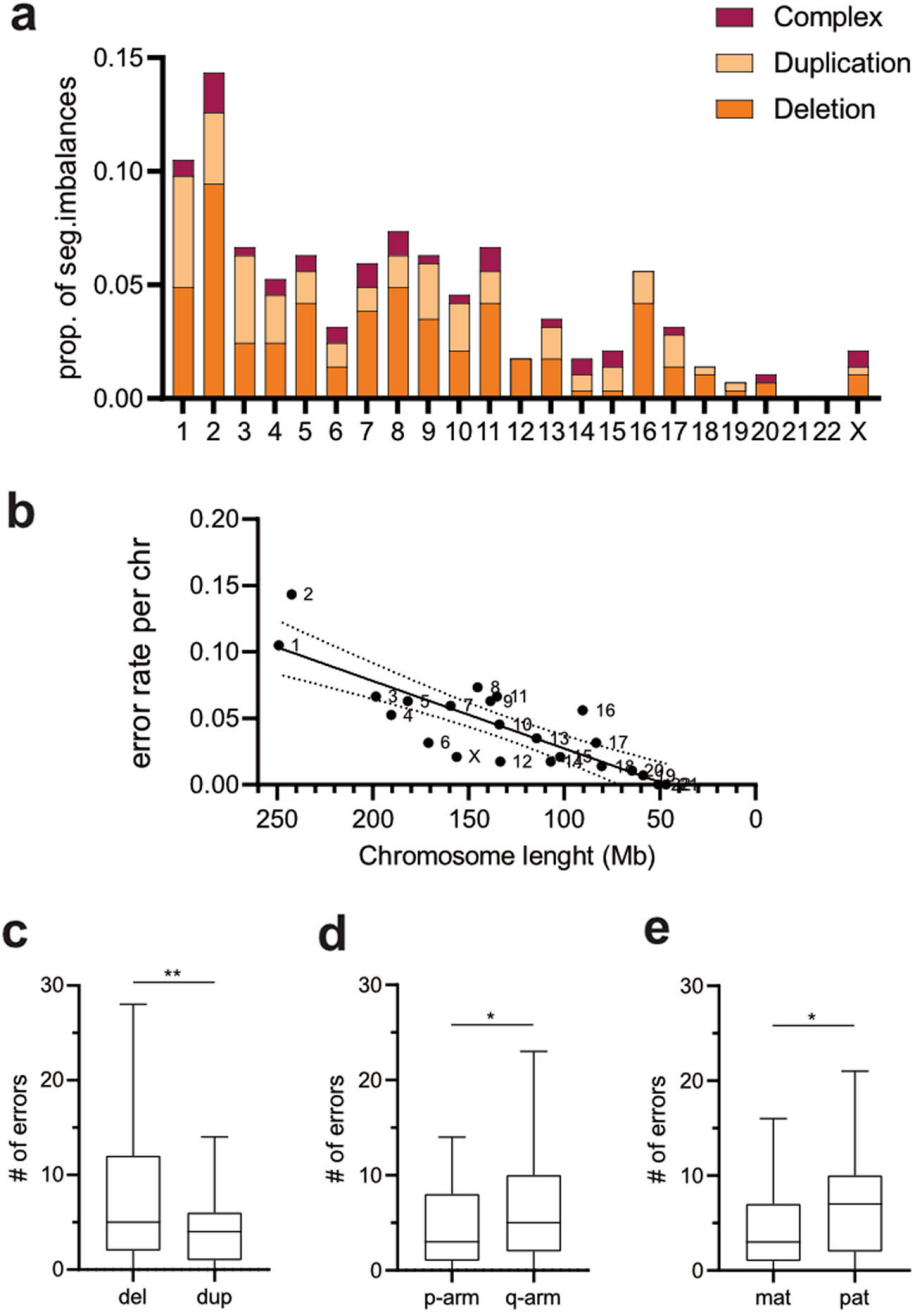
Segmental imbalances in cleavage embryos. **a** Rate and distribution of various types of segmental imbalances across the genome (*n* = 162, 91, and 41 for deletions, duplications and complex imbalances, respectively). **b** Correlation of chromosome-specific rate of segmental imbalances with chromosome length (*R*^2^ = 0.69, *P* < 0.0001). Boxplot comparison of simple segmental imbalances (*n* = 253) by nature (deletions vs. duplications) (**c**), location (p-arm vs. q-arm) (**d**) and parental origin (maternal vs. paternal) (**e**). Two-tailed *t*-test, **P = 0.008, *P = 0.02.

We next mapped the incidence and distribution of various segmental imbalances (≥10 Mb in size) in both D3 and D5/6 embryos (Fig. 4a). Recurrent meiotic segmental imbalances in embryos, derived from two PGT-SR families, were excluded, when characterizing segmental aneuploidy. In contrast to whole chromosome aneuploidy, segmental imbalances in D3 embryos rarely affect short chromosomes (chr19–22)^30,31^, which is also evident from the linear correlation between the chromosome length and the frequency of segmental aneuploidy (*R*^2^ = 0.69, *P* < 0.0001; Fig. 4b). However, small segmental imbalances ≤10 Mb fall below the single-cell aneuploidy detection limit, likely explaining the uneven distribution of segmental aneuploidy across the genome. We further classified segmental aneuploidy into (i) simple segmental imbalances with gains, losses or other rare aberrations either on p-arm or q-arm, and (ii) complex segmental imbalances, characterized by the presence of two or more aberrations on the same chromosome. In 80% of complex imbalances (*n* = 33/41), terminal deletion of one chromosome arm co-occurred with duplication of the other arm of the same chromosome. Intriguingly, paternal allele was affected in at least 2/3 of all complex abnormal imbalances (n = 29/41). Because the deleted and duplicated parts of the same chromosomes are derived from the same parent, they are likely to result from postzygotic chromosomal breakage and isodicentric chromosome formation. This configuration can consequently instigate breakage-fusion-bridge cycles, which result in one daughter cell with a single terminal deletion and one daughter cell with terminal deletion and an inverted duplication^32^.

For simple segmental imbalances (*n* = 253), deletions were more prevalent than duplications (*t* = 2.914, df = 22, *P* = 0.008; Fig. 4c) in our D3 dataset, and the long q-arm was more frequently affected by both duplications and deletions than the short p-arm (*t* = 2.489, df = 22, *P* = 0.02; Fig. 4d). Other segmental aberrations, such as segmental UPD or segmental nullisomy, were rare and each present with a frequency of less than 0.5% of all embryos. In contrast to whole chromosome aneuploidies where the majority were maternal in origin, segmental imbalances predominantly affected paternal chromosomes (*t* = 2.468, df = 22, *P* = 0.02; Fig. 4e). This was especially true for segmental deletions, with 61.5% being paternal origin (*n* = 162, *P* = 0.004, binomial test), but no such difference was observed for segmental duplications (*n* = 91, *P* = ns, binomial test). The same trend was observed in TE biopsies, as in 77% (*n* = 17/22) of segmental imbalances the paternal allele was missing. At the same time, more than half of sub-chromosomal rearrangements were mosaic, confirming the postzygotic mitotic nature of these segmental aneuploidies^1,30–32^.

## Discussion

Genome-wide embryo genotyping and haplotyping methods provide novel insight into the genome dynamics of preimplantation development. Here, we applied haplotyping to map the chromosomal constitution in cleavage and blastocyst stage embryos, derived from PGT-M patients. Overall, a single biopsy reveals that 43.2% and 24.9% of cleavage stage embryos and blastocysts, respectively, carry chromosomal abnormalities. This incidence is lower than previously reported in PGT-A studies^3,11,31,33^ in which the incidence is deduced in embryos, generated from couples with fertility issues. At the same time, the aneuploidy rate was significantly higher in cleavage stage embryos compared to blastocysts, which is consistent with previous reports. A study by Shahbazi et al.^24^ demonstrated that monosomies affect blastocyst expansion more severely than trisomies. This can also potentially explain the prevalence of monosomies in our D3, but not in TE data, as only good quality blastocysts were used for TE biopsy. In addition, cleavage biopsies often demonstrated more chaotic genome profiles, and 6% of all day-3 embryos had hypodiploid blastomeres, indicative of tripolar chromosome segregation. Most embryos with hypodiploid blastomeres did not arrest but progressed beyond cleavage stage, which suggests that tripolar mitosis occurred at 2nd or 3rd division, affecting only a part of the embryo. Moreover, clonal hypodiploid cells can be traced back in blastocysts, albeit these cells are seemingly excluded from the development prior to morula formation or during the morula-to-blastocyst transition^34,35^. Hence, our data supports the selective-pressure hypothesis against complex aneuploidy^11^, as hypodiploid or complex abnormal cells likely hit the transcription block upon EGA, thus failing to get incorporated into morula or blastocyst. Although euploid cells can ensure embryo development into good quality blastocyst, early tripolar division reduces the implantation potential of these embryos^27^.

The parent-of-origin analysis of segmental and whole chromosome aneuploidy revealed that segmental imbalances predominantly affected paternal chromosomes. This is opposite to whole chromosome, which was mostly maternal in origin. This observation contradicts an earlier study that analysed all blastomeres from a small set of embryos, suggesting paternal and maternal segmental imbalances occur with similar frequencies^32^; but is in line with two recent large scale studies on TE biopsies^19,36^. The paternal predominance was especially evident in deletions. Recently, single sperm sequencing revealed that meiotic segmental imbalances occur in only 0.4% of sperm cells^37^. Hence, the higher incidence of paternal segmental aberrations in embryos confirms their mitotic origin. Because duplications affect both parental alleles equally, they likely arise due to postzygotic non-disjunction, which occurs at a similar rate in maternal and paternal genome, generating reciprocal deletion/duplication in daughter blastomeres^1,4,5,30,32^. In turn, paternal segmental losses could originate from DNA damage in the sperm that can also trigger genomic instability in embryos^38^. However, considering that the analysis was done on a single D3 biopsy, the presence of reciprocal segmental imbalances in other blastomeres still cannot be excluded. At the same time, mature sperm cells lack DNA repair capacity, making them susceptible to DNA lesions, including double strand breaks (DSB), at later stages of spermatogenesis. Upon fertilization, sperm cells rely on maternal factors to repair their genome, mainly via error-prone non-homologous end joining (NHEJ) or homologous recombination^39^. However, if the DSBs remain unrepaired, they can lead to terminal loss of the acentric fragment. In some cases, truncated chromosomes can result in dicentric isochromosomes by replicated sister chromatid fusion. Consequently, chromosomes with inverted dup del, as well as pure terminal deletions are generated upon bipolar segregation^32^. Such sequence of events can also explain why the paternal allele was more frequently affected in embryos with complex rearrangements. Although we did not investigate the paternal age effect on the rate of segmental imbalances in our study, previously published studies did not reveal any association between the two^19,36^. Moreover, it remains unclear whether sperm damage or deficiency of maternal DNA repair genes can explain the presence of chromosomal rearrangements in human embryos or whether DNA breaks within the embryo can also occur during the first postzygotic divisions. Hence, further research is warranted to understand the true origin and clinical consequence of postzygotic segmental aberrations.

Our data demonstrates that parental origin of whole chromosome losses and gains in embryos correlates with mechanisms of aneuploidy formation. Single aneuploidy is primarily maternally driven due to meiotic errors in the oocyte. In contrast, mitotic errors showed no clear preference for either of parental alleles, confirming that post-zygotic aneuploidy does not discriminate between maternal and paternal homologs^11,40^. Moreover, mitotic aneuploidy was equally distributed across the whole genome. This is contradicting a previous report by McCoy et al., which suggested that putative mitotic errors are biased towards larger chromosomes^11^. However, this discrepancy can be a result of different sample size, as McCoy et al. explored a significantly larger D3 biopsy pool. At the same time, we confirm findings from other studies that chromosomes 15, 16, 19, 21, and 22 are more prone to maternal meiotic aneuploidy^11,24,41,42^. The prevalence of maternal MI errors in our study is also consistent with the recent analysis of TE biopsies, derived from PGT-M patients of similar age^19^. We also observed that acrocentric and/or short chromosomes are more susceptible to chromosome missegregation in meiosis I^43,44^. Alternatively, non-canonical reverse segregation may occur in the oocyte, in which sister chromatids are segregated at MI and homologous chromosomes at MII^43^. In combination with MII error, reverse segregation would result in the presence of two distinct maternal haplotypes, and thus classified as MI. The susceptibility of acrocentric chromosomes to meiotic errors is attributed to cohesion weakening and increased reverse segregation events in the oocyte that may predispose these chromosomes to aneuploidy^44^. Depending on chromosomes involved, aneuploidies can have variable impact on early embryo development: while trisomy 15 and 21 embryos initially develop similarly to euploid embryos, trisomy 16 impairs embryo growth already during early post-implantation period^24^. Remarkably, all maternal trisomy 16 cases were meiotic in origin. The surge of meiotic trisomy 16 in embryos together with its adverse impact on early development also explain why it is one of most frequently observed genetic abnormality in early spontaneous abortions up to 10 weeks of gestational age^45^, but is less commonly detected in non-invasive prenatal testing that is usually performed at week 12^46,47^.

Apart from whole and segmental aneuploidy, we observed genome-wide anomalies, such as triploidy or haploidy, in <1% of all cleavage and TE biopsies, which were mainly of maternal origin. Considering that oocytes were fertilized by ICSI, the prevalence of maternal meiotic errors in triploid and tetraploid cells can be expected, as ICSI precludes the formation of diandry via dispermic fertilization. Gynogenesis, on the other hand, may arise due to oocyte activation by the injected sperm that failed to decondense and replicate its genome. However, this mechanism does not explain the origin of the observed androgenetic blastomeres. Instead, extrusion of all maternal chromosomes and their spindles into polar bodies can underlie the formation of androgenetic zygotes^48^. Alternatively, heterogoneic division can lead to parental genome segregation in the zygote, giving rise to gynogenetic or androgenetic cells^49^. Albeit rare, embryos with genome-wide ploidy issues can grow and develop further, resulting in pregnancy complications and/or severe clinical phenotype in the fetus or newborn. Moreover, such aneuploidies are not detected by conventional PGT-A via low-pass sequencing. Hence, introducing genotyping and haplotyping-based embryo selection would enable ploidy abnormalities detection.

The main limitation of our study was the limited sample size of trophectoderm biopsies, thus correlation analyses were restricted due to lack of statistical power. Hence, more studies are warranted to understand the propagation of different types of aneuploidy throughout preimplantation period. In addition, application of haplotyping-based technology on individual cells of blastocysts can further unravel the true genomic landscape of embryos at later stages of development.

In conclusion, the current study complements the existing knowledge on the parental and mechanistic origin of whole chromosome and segmental aneuploidy in preimplantation embryos. Our results implicate different mechanisms that can predispose embryos to chromosome segregation errors. The ability of embryos with complex postzygotic aneuploidy to bypass the developmental arrest also raises an important questions about the potential selective mechanisms that might operate in human embryos without compromising its survival.

## Methods

### Study design

This is a retrospective study that was approved by the Ethical Committee of UZ/KU Leuven (S63000). The study was conducted in compliance with the principle of Declaration of Helsinki and GDPR (General Data Protection Regulation 2016/679), and in accordance with all applicable regulatory requirements. All patients received information on the study and provided informed consent on the use of their data. We analysed accumulated embryo data from families that enrolled in haplotyping-based PGT-M program at UZ Leuven between 2014 and June 2020. The PGT-M cohort includes a presumably fertile population with maternal age ranging from 20 to 42 (mean maternal age at the start of first PGT cycle was 30.11 ± 3.95). In total, 405 couples had indication for PGT-M and 11 for PGT-SR for structural rearrangements, resulting in 2618 informative embryos available for analysis (2257 cleavage-stage and 361 blastocyst stage embryos, respectively).

### Embryo culture and biopsy

A standard clinical workflow for PGT-M was performed at UZ Leuven^50^, AZ Sint-Jan Brugge-Oostende AV and UCLouvain. Oocytes were fertilized by intracytoplasmic sperm injection (ICSI), followed by embryo culture. Both cleavage stage (UZ Leuven and UCLouvain) and TE biopsies (UZ Leuven and AZ Sint-Jan) were performed. All oocytes and embryos were cultured at 37 °C in 5–6% CO_2_ and 5% O_2_ in single Global Total^®^ or Global Total LP^®^ medium (CooperSurgical, USA) under mineral oil (UZ Leuven) or G-TL™ medium (Vitrolife, Sweden) (AZ Sint-Jan). All day-3 cleavage stage embryos that had ≥6 blastomeres were subjected to laser-mediated embryo biopsy using Saturn 5^TM^ Laser system (CooperSurgical, USA), which was performed in Ca2+/Mg2+ free medium (Global, LifeGlobal^®^, Origio, Benelux) at UZ Leuven or HAS/G-PGD medium (Vitrolife, Sweden) at UCLouvain. One blastomere was biopsied, except in rare cases, when two cells were removed from the embryo. Following biopsy, embryos were either vitrified (UCLouvain) or cultured further until blastocyst stage (UZ Leuven). For UZ Leuven, day-3 biopsies were analysed only if biopsied embryos developed into blastocysts and were vitrified (exceptionally, a subset of biopsies were processed immediately, e.g., in case of foreseen fresh embryo transfer). If the cleavage-stage sample failed to deliver a qualitative result, a re-biopsy was performed at blastocyst stage, removing 5–10 trophectoderm cells from the embryo. For fresh TE biopsy in both UZ Leuven and AZ Sint-Jan, laser assisted zona opening was first performed on day 3. Depending on blastocyst development, the biopsy was done on days 5–7 of post-insemination, aspirating approximately 5–10 cells with laser pulses (Saturn 5^™^ Laser system) in combination with mechanical detachment (flicking). The biopsy was performed in Global, LifeGlobal^®^ medium (UZ Leuven) or G-MOPS^™^ PLUS medium (Vitrolife, Sweden) at AZ Sint-Jan, overlaid with paraffin culture oil (Ovoil^™^, Vitrolife, Sweden). All biopsied cells were rinsed in 1% PVP/PBS droplets, transferred into PCR tubes filled with 2 μl PBS and kept frozen until further processing.

### Biopsy processing

All biopsied samples were whole-genome amplified (WGA) by multiple displacement amplification using REPLI-g Single Cell kit (Qiagen, Germany), according to manufacturer’s instructions, but with reduced incubation time of 2 h and inactivation of the enzyme at 65 °C for 3 min. Following WGA, successfully amplified samples were genotyped using Illumina HumanCytoSNP-12 BeadChip. Parental and first-degree relatives (i.e., parents of prospective parents or a couple’s offspring) bulk DNA, extracted from blood, was also genotyped for subsequent haplotyping analysis.

### Genome-wide embryo profiling using haplarithmisis

Embryo PGT-M data analysis was performed using siCHILD/haplarithmisis^14^, which exploits SNP genotypes and SNP B-allele frequencies (BAF) to reconstruct genome-wide haplotypes and map genomic aberrations. Karyotype information was retrieved for all previously analyzed embryos. Embryo biopsies with low data quality, resulting in inconclusive haplarithmisis result, were excluded from this study. All genetic abnormalities present in the rest of the embryos were divided into whole-chromosome (e.g., monosomy, trisomy, and nullisomy), segmental (chromosomal losses and gains >10 Mb) and genome-wide abnormalities (e.g., haploidy, triploidy). With the exception for monosomies, the use of SNP BAF-values, which are segmented into parental haplotype blocks, allows to distinguish meiosis I (MI), meiosis II (MII), and postzygotic mitotic trisomy. Hence, whenever possible, parental and mechanistic origin (MI, MII, or mitotic error) of genomic abnormality were also recorded.

### Blastocyst disassociation for single-cell analysis

To test the hypothesis that tripolar mitosis in cleavage stage embryos occurred after the first zygotic division, four blastocysts were thawed and disassociated for single-cell analysis. For manipulations of the whole blastocyst a STRIPPER pipette with 175 or 135 µm capillaries (Origio, CooperSurgical, Inc., USA) was used. The dissociation procedure was performed as follows: removal of the zona pellucida was obtained by short incubation of the blastocyst in Acidic Tyrode’s solution (Sigma-Aldrich, Merck KGaA, Germany) until visual disappearance of the zona pellucida was observed. The blastocyst was consecutively washed in three drops of biopsy medium (LG PGD Biopsy Medium, Life Global) and incubated in trypsin at 37 °C. Subsequently, the blastocyst was washed three times in biopsy medium. Individual cells or clumps of 2–3 cells from the blastocysts were then isolated by manual pipetting using a STRIPPER pipette with a 75 µm capillary (Origio, CooperSurgical, Inc., USA) and washed three times 1% PVP-PBS. Subsequently, each isolated cell(s) was transferred into a 0.2 ml PCR tube with 2 µl PBS and stored at −20 °C until further use. Samples were then whole genome amplified using REPLI-g Single Cell Kit (Qiagen, Germany) according to the protocol mentioned above with incubation at 30 °C for 2 h followed by 65 °C during 10 min for inactivation.

### Statistical analysis

All statistical analysis was performed using GraphPad Prism (San Diego, USA, version 6). A two-tailed Fisher’s exact test was used to compare the categorical data between two different developmental stages. Binominal test was used when comparing two possible outcomes, such as maternal/paternal or mechanistic origin of aberration. Linear regression was used to evaluate the association between the rate of segmental imbalances and chromosome length. Two-tailed *t*-test was used to assess the location, type and parent-of-origin of segmental aberrations across the genome.

### Reporting summary

Further information on research design is available in the Nature Research Reporting Summary linked to this article.

## Supplementary information

Supplementary Information

Reporting Summary

## Data Availability

In compliance with the GDPR, the dataset used in the study is not publicly available. Embryo, parental and phasing relatives raw genotyping data is available to academic users upon request to the Data Access Committee (DAC) of KU Leuven via the senior co-authors (J.R.V. and E.D.).
