# Supplementary material for: Haplotyping-based preimplantation genetic testing reveals parent-of-origin specific mechanisms of aneuploidy formation
Source: NPJ Genom Med. 2021 Oct 7;6:81. doi: 10.1038/s41525-021-00246-0 (PMC8497526; doi:10.1038/s41525-021-00246-0)
Supplement: Supplementary file 1 — Supplementary Information [file 41525_2021_246_MOESM1_ESM.pdf]

Supplementary Material

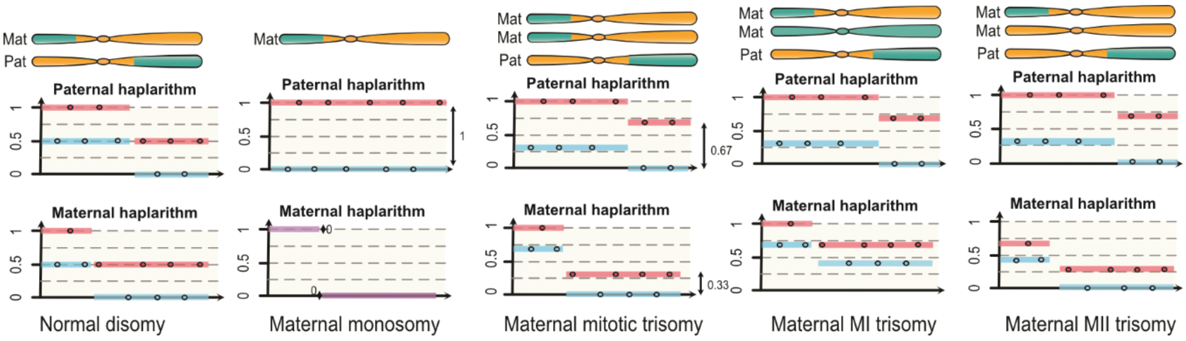

Supplementary Figure 1. Schematic representation of haplarithm plots. Genome-wide haplarithm profiles for normal disomy, maternal monosomy and maternal trisomy. Based on haplotypes and recombination patterns, meiosis I (MI), meiosis II (MII) and mitotic aneuploidy can be defined. If signatures of monosomy or trisomy occur across all chromosomes, it indicates haploidy or triploidy, respectively.

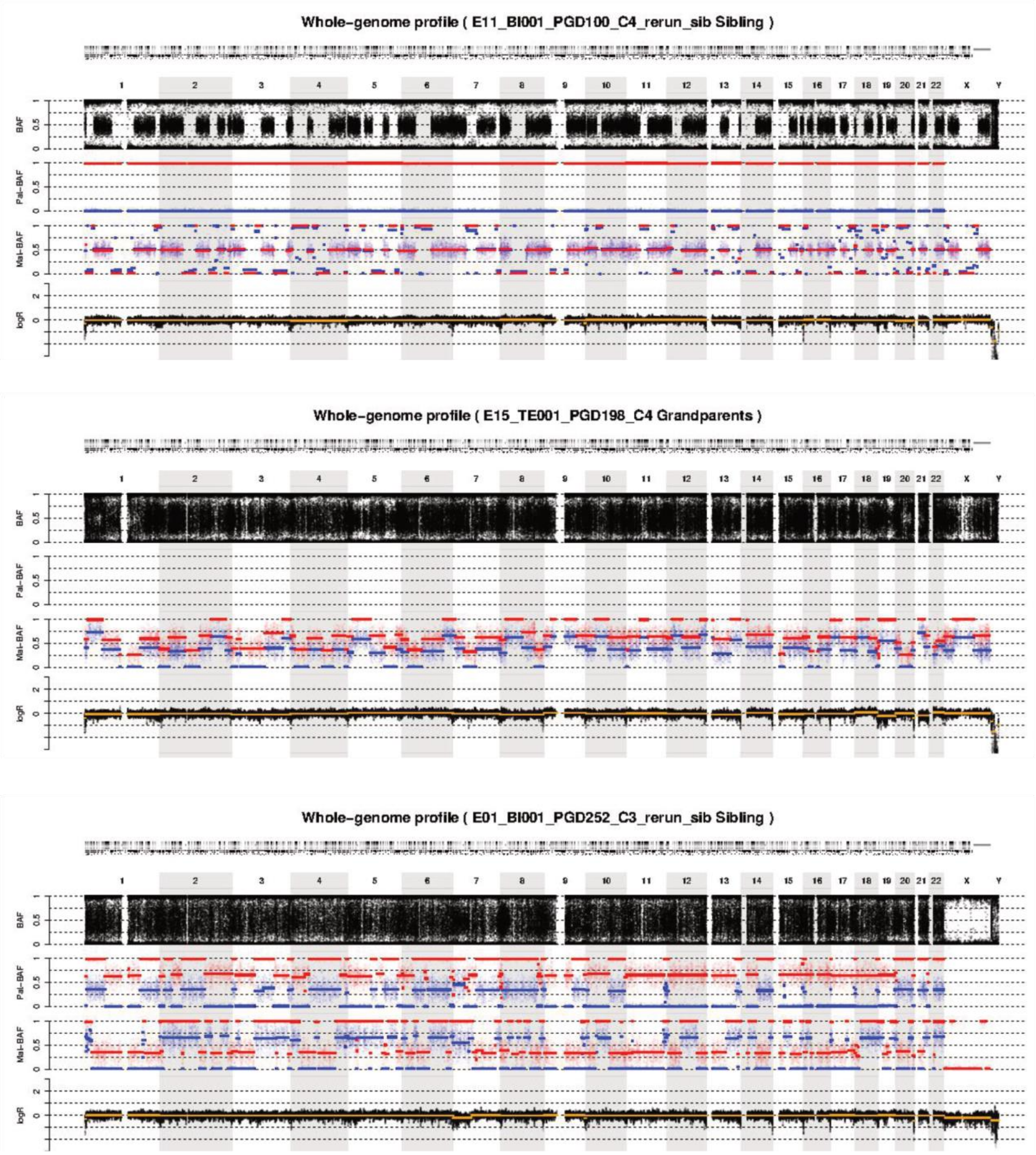

Supplementary Figure 2. Genome-wide ploidy aberrations in blastocysts. Haplarithm profiles depicting likely parthenogenetic blastocyst **(top)**, digynic triploid with signatures of meiosis I error (MI)/polar body non-extrusion **(middle)** and digynic triploid with signatures of mitotic chromosome-missegregations genome wide **(bottom)**. For E11(top) and E15 (middle) parents of the mother (maternal grandparents) were used for phasing, providing maternal haplarthm profiles (Mat-BAF). For E01 (bottom), an affected offspring was used for phasing, thus both maternal (Mat-BAF) and paternal (Pat-BAF) haplarithms are available.

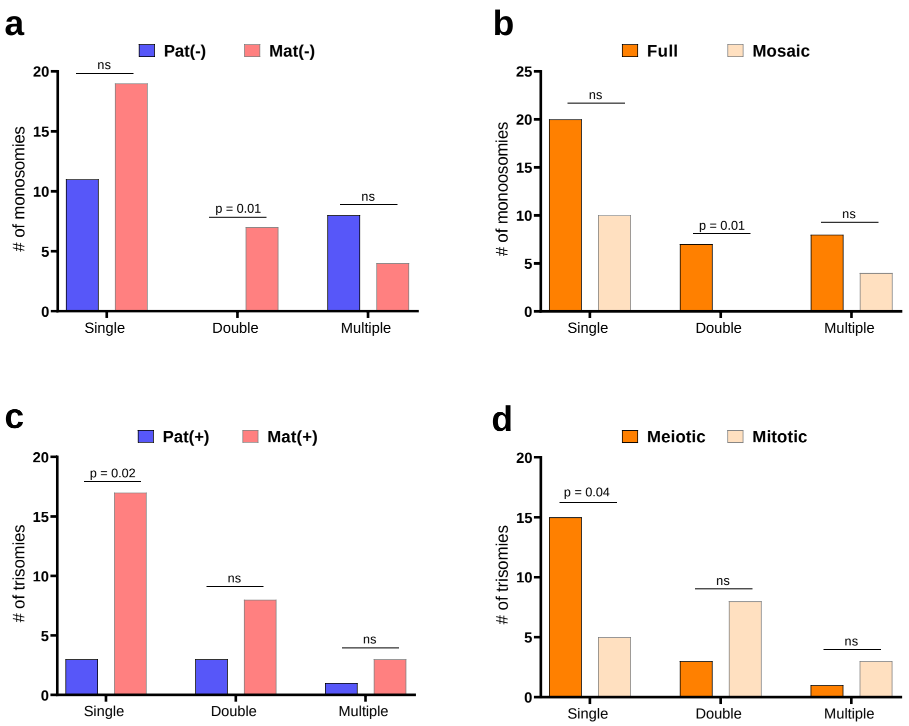

Supplementary Figure 3. Parental and mechanistic origin of aneuploidy in blastocysts. Number of monosomies with parental **(a)** and mechanistic **(b)** origin; n = 30, 7 and 12 for single, double and multiple (3-5 chromosome affected), respectively. Number of trisomies with parental **(c)** and mechanistic **(d)** origin; n = 20, 11 and 4 for single, double and multiple (3-5 chromosome affected), respectively. Two-tailed binomial test was used for statistical analysis.

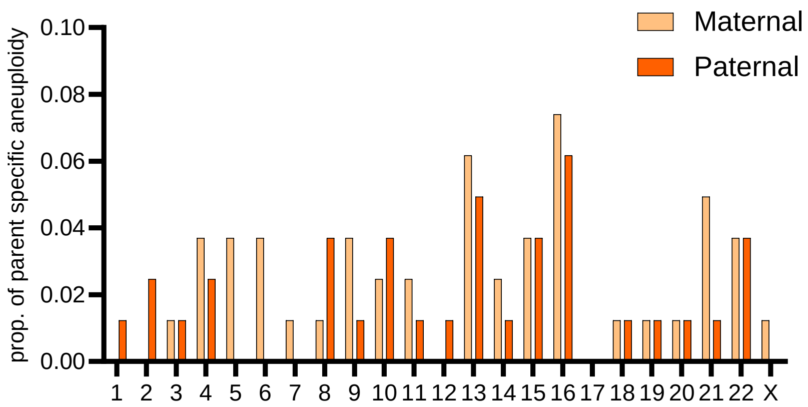

Supplementary Figure 4. Aneuploidy distribution in blastocysts. Depicted is whole chromosome aneuploidy (n = 84) rate across the genome in blastocyst, based on a single TE biopsy.

Supplementary Table 1. Blastocyst morphology and grade, following putative tripolar division at cleavage stage

Embryo ID	Chr affected	Blastocyst morphology D5	Overall blastocyst score
PGD036_C4II_E13	15	Expanding blastocyst BB1 + loose cells in ICM	Average
PGD037_C3_E09	16	Very early blastocyst	Poor
PGD037_C5_E05	13	Expanding blastocyst + cell out	Poor
PGD048_C3_E02	19	Very early blastocyst + cell out	Poor
PGD054_C2_E02	12	Arrested	Arrested
PGD062_C2_E01	12	Very early blastocyst out of ZP / large cells left in ZP	Poor
PGD068_C3_E03	20	Very early blastocyst	Poor
PGD078_C3_E05	14	Very early blastocyst + cell out	Poor
PGD090_C1_E05	16	Hatched blastocyst AA1	Excellent
PGD093_C2_E01	17	Early blastocyst BB2	Poor
PGD099_C1_E08	14	Expanding blastocyst BB2	Poor
PGD101_C2_E01	20	Expanding blastocyst BB2	Poor
PGD114_C4_E07	21	Expanding blastocyst, BB3	Poor
PGD116_C2_E05	15	Very early blastocyst + cells out	Poor
PGD118_C2_E12	22	Early blastocyst	Average
PGD120_C1_E01	13	Arrested	Arrested
PGD127_C4_E11	15	Expanding blastocyst, BB2	Poor
PGD133_C1_E01	14	Expanded blastocyst, AB1	Excellent
PGD139_C1_E06	12	Hatching blastocyst, AA1	Excellent
PGD148_C1_E07	18	N/A	N/A
PGD148_C1_E14	20	N/A	N/A
PGD148_C1_E03	21	N/A	N/A
PGD148_C1_E05	18	N/A	N/A
PGD158_C1_E10	16	Hatching blastocyst, AB2 + loose cell	Good
PGD159_C2_E09	13	Hatching blastocyst, AC1	Average
PGD164_C1_E12	18	Very early blastocyst + cells out	Poor
PGD170_C2_E17	17	Arrested	Arrested
PGD172_C2_E02	20	Early blastocyst	Average
PGD181_C1_E03	16	Very early blastocyst	Poor
PGD184_C1_E03	14	Hatching blastocyst, BA2	Good
PGD187_C1_E09	16	Hatching blastocyst, AA2	Good
PGD202_C1_E04	15	Arrested	Arrested
PGD203_C1_E04	18	Arrested	Arrested
PGD203_C1_E05	14	Arrested	Arrested
PGD205_C3_E18	17	Early blastocyst	Average
PGD210_C2_E05	16	Early blastocyst + cells out	Poor
PGD226_C3_E03	21	Hatching blastocyst, AB1	Excellent

PGD238_C1_E05	13	Arrested	Arrested
PGD246_C1_E09	20	Early blastocyst + cells out	Poor
PGD246_C1_E14	12	Very early blastocyst	Poor
PGD248_C3_E03	17	Very early blastocyst + cells out	Poor
PGD300_C3_E03	12	Hatching blastocyst, BB1 + cells out	Average
PGD310_C1_E04	13	Early blastocyst	Poor
PGD313_C4_E05	13	Very early blastocyst + cells out	Poor
PGD323_C1_E07	15	Very early blastocyst	Poor
PGD326_C2_E07	22	Very early blastocyst + cells out	Poor
PGD333_C1_E05	16	Expanding blastocyst, BB2	Poor
PGD333_C1_E08	16	Expanding blastocyst, BC2	Poor
PGD334_C3_E03	12	Very early blastocyst + cells out	Poor
PGD336_C3_E02	15	Very early blastocyst	Poor
PGD343_C1_E06	17	Expanding blastocyst, BB2	Poor
PGD343_C1_E11	14	Early blastocyst	Poor
PGD344_C3_E01	21	Very early blastocyst + cells out	Poor
PGD345_C1_E06	12	Very early blastocyst + cells out	Poor
PGD347_C4_E13	14	Expanding blastocyst, AB2 + frag	Average
PGD347_C4_E11	15	Hatching blastocyst, BB2 + frag	Average
PGD384_C1_E13	16	Expanding blastocyst, BB1 + large cell out	Average
PGD385_C1_E03	17	Expanding blastocyst, BA1	Good
PGD511_C1_E02	15	Very early blastocyst + frag	Poor
PGD514_C1_E01	12	Arrested	Arrested
PGD514_C1_E04	13	Arrested	Arrested
PGD546_C2_E06	18	Expanding blastocyst, BB1	Average
PGD579_C1_E01	15	Expanding blastocyst, BB1	Average
